# The Beat

**Published:** 2008-11

**Authors:** Erin E. Dooley

## Green Energy Credit Renewed

The renewable energy industry held its breath as Congress decided whether to renew tax credits for residential and commercial use of alternatives such as wind, solar, and tidal power [see “Solar Tax Credit Loses Energy,” *EHP* 116:A380 (2008)]. Now the decision has been made: the $18 billion plan was signed into law 3 October 2008 as part of the Emergency Economic Stabilization Act of 2008. Under the plan, credits for solar energy are extended for 8 years, those for wind energy for 1 year, and those for other renewable energy sources including wave and tidal energy for 2 years. The bill also eliminates a $2,000 cap on residential solar systems and includes an allowance for utilities to use the commercial credit. In addition, the legislation allows $800 million in bonds to finance renewable energy facilities and provides $1.5 billion in tax credits for carbon capture-and-storage projects.

**Figure:**
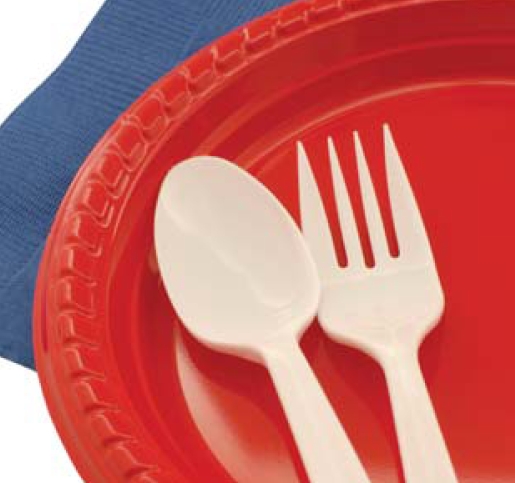


## France Scraps “Picnic Tax”

On 15 September 2008 French environment minister Jean-Louis Borloo announced the country would institute a “picnic tax” on disposable paper and plastic plates, cups, and cutlery as part of a package of measures aimed at reducing waste and energy use. Just 3 days later, prime minister François Fillon threw out the tax in the face of overwhelming negative response from the public and political opposition, who called the measure an unfair burden on ordinary citizens enjoying a popular French pastime. Similar taxes said to be still under consideration apply to other high-volume consumer products such as batteries, televisions, and appliances, with consumers purchasing environmentally friendly versions of these products seeing tax breaks.

## Nitrate Sampled in U.S. Wells

Since the mid-20th century, use of nitrate fertilizer has steadily increased in the United States. One of the first nationwide studies of nitrates in groundwater over time, published in a September 2008 supplement to the *Journal of Environmental Quality*, has found that nitrate concentrations increased significantly between 1988 and 2004 in almost a third of well networks sampled. In three networks, median concentrations exceeded the EPA maximum contaminant level of 10 ppm, reaching 11.68 ppm in the Delmarva Peninsula. Nitrate, a common groundwater contaminant, has been linked in infants to methemoglobinemia, caused by reduced ability of blood to carry oxygen.

## Triple Washed, Twice Shy?

**Figure:**
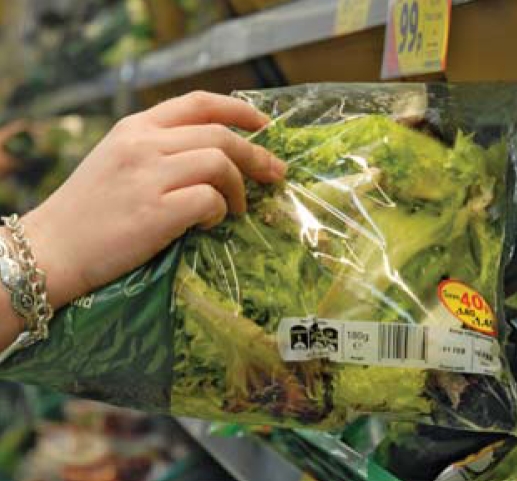


Consumers often look to convenient forms of produce to put more vegetables in their diet. An expert on foodborne pathogens speaking at the Food Micro 2008 conference in September suggested consumers should wash prepacked produce even though labels claim bagged vegetables are “ready to eat.” Gad Frankel of Imperial College London pointed out that pathogens such as *Salmonella* and *E. coli* can still bind to vegetable leaves. In research published 2 October 2008 ahead of print in *The ISME Journal*, Frankel described how *S. enterica* uses its flagella to cling to basil leaves, a finding that may offer clues for enhancing food safety.

## Bacterium Is Arsenic Watchdog

A newly discovered “extremophile,” a bacterium that thrives at ultracold temperatures, not only remediates arsenic-contaminated sites but may also serve as a living sensor to warn of arsenic escaping from mines and chemical facilities and to test well water. Discovered in northern Canada’s Giant Mine, the bacterium lives in communities of biofilms where it converts arsenite, an intractable form of arsenic, into arsenate, which is relatively easy to remove. Thomas Osborne and colleagues from University College London announced their finding at the Autumn 2008 meeting of the Society for General Microbiology.

## Arab Cities Get Green Standards

Ahead of the first Eco-Cities of the Mediterranean Forum, an October 2008 meeting on urban sustainability, Jordanian environment minister Khaled Irani announced a new initiative to set standards for renewable energy, carbon emission reductions, and environmental policy in Mediterranean cities. Jordanian officials organized the forum, which they hope will become a biennial event, to encourage partnerships and to learn from the experience of more than 200 global experts and policy makers in addressing the environmental challenges and opportunities facing the region.

**Figure:**
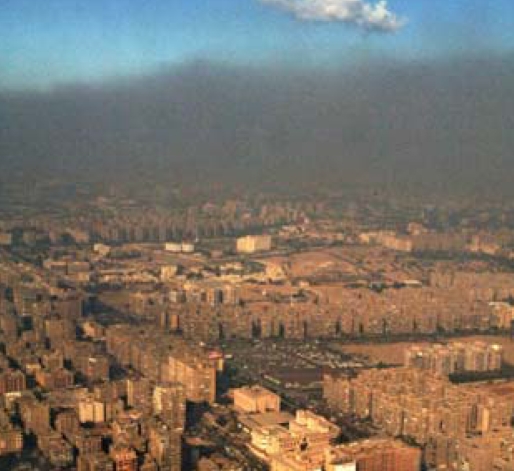
Smog blankets Cairo, October 2006

